# Frying Conditions, Methyl Cellulose, and K-Carrageenan Edible Coatings: Useful Strategies to Reduce Oil Uptake in Fried Mushrooms

**DOI:** 10.3390/foods10081694

**Published:** 2021-07-22

**Authors:** Montserrat Martínez-Pineda, Cristina Yagüe-Ruiz, Antonio Vercet

**Affiliations:** Faculty of Health and Sports Science, University of Zaragoza, 22002 Huesca, Spain; cyague@unizar.es (C.Y.-R.); vercet@unizar.es (A.V.)

**Keywords:** methyl cellulose, kappa-carrageenan, edible film, oil uptake, frying, coating

## Abstract

Despite being widely consumed and appreciated, fried food has the unhealthy characteristic of high final oil content. Therefore, alternatives to reduce the oil uptake of fried products are being researched. The aim of this study was to investigate the effect of 0.5% methyl cellulose and 0.5% kappa-carrageenan edible films, as well as different frying procedure parameters, such as oil temperatures (from 150 to 180 °C), and thickness of slices (from 2 to 6 mm) on the oil uptake of whole fried mushrooms and their parts. The results showed a lower final oil content when lower frying temperature and thicker slices are applied. Hydrocolloid suspensions of methyl cellulose and kappa-carrageenan, used as edible coatings, were effective at reducing moisture evaporation and, consequently, oil uptake independently of the hydrocolloid temperature. A reduction of 10–22% in the final oil content was achieved. Adjusting the frying parameters and the use of methyl cellulose or kappa-carrageenan as an edible coating were useful strategies to reduce the oil uptake in fried products.

## 1. Introduction

Deep fat frying involves cooking food by immersion in edible oil or fat at a temperature above the boiling point of water, where the fat serves as the heat transfer medium. As a consequence, there is a mass transfer whereby water and other soluble materials are transferred from the food being fried as the oil penetrates the product, generally resulting in food with a high fat content [1,2].

In recent years, due to the unhealthy implications of this type of cooking method, especially in relation to obesity and cardiovascular diseases [3], many innovative solutions to decrease the fat content of fried products have been proposed. Some of the alternatives to reduce oil uptake are associated with new frying technologies, such as pressure frying [4], microwave frying [5], or vacuum frying [6]. Another approach to reduce oil uptake is the use of different batter and coating systems such as oil barriers [7]. Edible coatings, defined as a thin layer of edible material formed as a coating on the product, act as a blocker to oxygen, moisture, and solute movement for food without changing its original ingredients, and usually have to be as tasteless as possible in order not to be detected during consumption [8]. They are applied as liquids on the food surface, generally by spraying, dipping, a fluidized bed, or panning, but also by electrospraying [9,10,11]. Several coating formulations have been studied, lipid-based and hydrocolloid-based (proteins and polysaccharides), with different objectives from protecting food from microbial growth and prolonging their shelf life to improving their sensory attributes [12]. Hydrocolloid coatings have been widely investigated due to their thermal gelation and thickening properties as alternatives for oil uptake reduction in deep-fat-fried foods. Protein-derived edible coatings, such as corn zein, muscle, soy, or whey protein, among others, were effective for this purpose [13]. Polysaccharide-based coating cellulose and derivatives, such as methylcellulose, pectins, starch, and certain gums, have been studied in fried potato, chicken, and cereal products [7,14]. Plasticizers such as glycerine, propylene glycol, and sorbitol are also frequently added by researchers as ingredients in edible coatings [7,15]. 

It should be remarked that the majority of the studies that analysed frying oil uptake, as well as polysaccharide-based edible coatings for reducing oil uptake, have focused on fried potato, chicken, and cereal products. Studies have rarely been performed on vegetable products with spongy structures such as mushrooms.

Therefore, the aim of this study was to investigate the effect of methyl cellulose and kappa-carrageenan edible films, as well as different frying procedure parameters, oil temperatures, and thickness of slices, on the oil uptake of fried mushrooms.

## 2. Materials and Methods

### 2.1. Material and Hydrocolloid Suspension Preparation

This study was carried out using fresh mushrooms (Agaricus bisporus) (4.6 ± 1.6 mm diameter). As hydrocolloids, methyl cellulose (MC) and K-carrageenan (K-c) (Texturas- Ferrán and Albert Adriá) were studied, provided by Bidfood Iberia (Barcelona, Spain). High-oleic sunflower oil (Titan, Koipe, S.A., Gipuzkoa, Spain), purchased from a local supermarket, was used for frying.

### 2.2. Hydrocolloid Coating Preparation 

Two different edible coatings were analysed: a 0.5% aqueous MC suspension and 0.5% aqueous K-c suspension. MC and K-c edible coatings concentrations usually range between 1–2%, however the effect of lower concentration (0.5%) was analysed in this study. Plasticizers were used in none of the edible coating formulations. Dry hydrocolloid was added to water and allowed to dissolve with mixing using a kitchen mixer (Moulinex, Groupe Seb Ibérica, Barcelona, Spain). 

### 2.3. Coating Rheology Characterization

Before calculating batter pick-up, the flow behaviour of each hydrocolloid suspension was analysed. Flow behaviour was analysed immediately after the hydrocolloid suspensions were prepared, using a cone and plate rotational viscometer (Brookfield’s Ametek CAP-2000 +, Middleboro, MA, USA). It was equipped with a Peltier thermoelectric controller and the software CAP-CALC/CAP266Y (v. 3). We used a linear ramp varying from 533 to 1333/s for the MC 30, MC 40, and K-c 30 samples, and from 667 to 1467/s for the MC 15 and K-c 40 samples; assays were performed at 30 °C. The shear stress/shear rate data were plotted as flow curves and fitted to the Power Law model. The shear stress, shear rate, as well as apparent viscosity, were calculated. Three experimental runs were performed for each assay replication.

### 2.4. Sample Preparation and Frying Conditions 

The effects of frying temperature and slice thickness on the oil uptake of uncoated mushrooms were studied. The effect of frying temperature was studied using mushroom slices that were 4.0 ± 0.2 mm thick and were fried for 150 s at 150 °C, 170 °C, or 180 °C. The effect of slice thickness was analysed by slicing mushrooms to a thickness of 2.0 ± 0.2 mm, 4.0 ± 0.2 mm, and 6.0 ± 0.2 mm using a slicing machine (CF4821, UFESA, Barcelona, Spain) and frying them for 150 s at 170 °C. 

To analyse oil capture kinetics during frying at 170 °C, uncoated mushroom slices (4.0 ± 0.2 mm thick) were fried and then removed from a deep-fat fryer after 30, 60, 90, 120, or 150 s.

For coated samples, mushroom slices (4.0 ± 0.2 mm thickness) were dipped into the hydrocolloid suspension for 10 s and allowed to drip for 12 s before frying. Different temperatures of edible coatings were tested. During the dipping process, methyl cellulose suspensions were maintained at 15 °C (MC 15), 30 °C (MC 30), and 40 °C (MC 40), while K-carrageenan suspensions were at 30 °C (K-c 30) and 40 °C (K-c 40). The coated mushroom slices were then fried for 150 s at 170 °C.

In all cases, a 3-L deep-fryer (Magefesa, Rhointer España, Santander, Spain) was used, and only two coated slices were fried at the same time. Each assay was performed in triplicate.

### 2.5. Coating Pick-Up Determination

The coating pickup was calculated to determine the amount of batter that adhered to the piece of food; this was a conditioning factor for the final product characteristics. The edible coating pick-up was calculated as follows:

Batter pick-up = [(CW − IW)/IW] × 100,(1)

where CW is the weight of the coated mushroom slice after dipping and IW is the initial weight of the uncoated mushroom slices. The determination was made in 10 slices per each assay replication (n = 30). Those samples were discarded after pick-up determination.

### 2.6. Moisture Content 

For moisture determination, 15 g of the sample were weighed in a glass evaporating basin and dried in a laboratory-drying oven (BINDER ED53, BINDER GmbH, Tuttlingen, Germany) at 105 °C until constant weight. The moisture content was calculated from the difference in weight before and after oven drying. These samples were then used to determine the oil content. Results were expressed as a percentage.

### 2.7. Oil Content

In order to determine the oil content, the dried and crushed samples were subjected to petroleum ether (Panreac Química S.L.U, Barcelona, Spain) extraction using FOSS Soxtec 2055 equipment (Foss Analytical, Hilleroed, Denmark). The fat content was determined after the extraction by weight difference [16]. The results were expressed as a percentage and calculated as:

Oil content = (OW/FW) × 100,(2)

where OW is the weight of extracted oil and FW is the weight of the fried sample.

In each assay replication, the moisture and fat content of the sample obtained were analysed in triplicate (n = 9).

### 2.8. Frying Oil Control Parameters

As fried foods often exhibit altered physicochemical values when they are fried under suboptimal conditions, such as when the oil is highly degraded [17], the physicochemical characteristics of the frying oil were analysed after each assay and the oil was changed when necessary to avoid interferences due to the degradation of compounds. 

#### 2.8.1. Polar Compounds

Measurement of polar compounds (in percentage) was done using a Testo 265 oil sensor (Instrumentos Testo S.A., Cabrils, España) at the end of each frying cycle. This rapid method, based on dielectric constant measurements, has been validated by [18] using thin-layer chromatography as a reference method.

#### 2.8.2. Oil Viscosity

A rotational cone-plate viscometer Brookfield’s CAP-2000 + (Brookfield’s Ametek CAP-2000 +, Middleboro, USA) equipped with a Peltier thermoelectric controller and the software CAP-CALC/CAP266Y (v. 3) was used to measure the different oil viscosities. A ramp temperature varying from 25 °C to 75 °C at 107 1/s of shear stress was applied.

#### 2.8.3. Iodine Value

The iodine value was determined according to Wijs, as indicated by the AOAC Official Methods of Analysis (1984) [19]. Oil samples (0.5–0.6 g) were placed in a 300-mL flask and dissolved in 15 mL of carbon tetrachlorine. Then, 25 mL of Wijs solution were added and the mixture was gently stirred. It was left to stand in the dark for 1 or 2 h, depending on the expected iodine value of the sample. Then, 20 mL KI solution (10%) and 150 mL of water were added. The excess iodine was titrated with 0.1 N Na2S2O3 with constant and vigorous shaking. When the yellow colour almost disappeared, 1 mL of soluble starch (1%) solution was added until the blue colour disappeared.

#### 2.8.4. Conjugated Dienes and Trienes 

The method described by Matissek et al. [20] was used to determine the presence of conjugated di- and triene groups. Oil samples (0.05 g) were placed in a 20-mL volumetric flask and the total volume was adjusted by adding isooctane.

A reference solution, consisting of methyl stearate 1% in isooctane, was also prepared. The spectrum of the sample was read against the blank at λ = 236 nm and 270 nm, respectively. Absorption was measured with a Jasco UV-Vis Spectrometer V-530 (Easton, MD, USA), controlled by a personal computer with the software Spectra Manager.

### 2.9. Fatty Acids Profile

The fatty acid composition of the frying oil and fried mushroom oil extracted were determined according to the EU Regulation 796/2002 (Commission Regulation (EC) No 796/2002) [21]. First, 2.0 mL of hexane was added to 0.1 g of sample and vigorously mixed. Then, 0.2 mL of potassium hydroxide methanolic solution (0.2 N) was added, vigorously mixed, and then the solution was left in the dark for 15 min. Next, 0.5 mL of the top part of the solution was placed in a vial, and 0.02 mL of internal standard was added. Fatty acid methyl ester (FAME) separation was performed in a gas chromatograph Hewlett-Packard HP5890 series II (Hewlett-Packard, Spring, TX, USA) with a split/splitless injector and an FID detector, equipped with a HP-88 capillary column of 100 m length, 0.25 mm i.d., and 0.20 μm film thickness (J & W Scientific, Folsom, CA, USA). The temperature program followed these steps: initial temperature, 120 °C (hold for 1 min); from 120 °C to 175 °C at 10 °C/min (hold for 10 min); from 175 °C to 210 °C at 5 °C/min (hold for 5 min); from 210 °C to 230 °C at 5 °C/min, then hold at 230 °C for 30 min; run time, 62.50 min. The injector and detector temperatures were 250 °C and 280 °C, respectively. Hydrogen was used as a carrier gas at a flow rate of 1.18 mL/min. A split ratio of 43:1 was used and 1.0 μL was injected in GC for the analysis. Data acquisition and processing were performed with Hewlett-Packard Chemstation software. FAME were identified by comparing their retention times with those for commercial standard mixtures (Supelco 37 component FAME Mix, linoleic acid methyl ester isomer mix, and linolenic acid methyl ester isomer mix; Supelco, Bellefonte, PA, USA). Methyl tridecanoate (puriss, GC grade, Sigma-Aldrich Chemical Co., Steinheim, Germany) was used as an internal standard.

### 2.10. Statistical Analysis

The results of rheology behaviour, pick-up, moisture, and lipid content were subjected to one-way ANOVA followed by Tukey’s post hoc test, the level of *p* < 0.05 being considered significant. Statistical analyses were performed with GraphPad Prism 5 (GraphPad Software, Inc., San Diego, CA, USA). Rheological characterization results were fitted to a Power-law rheological model while adjusting to R2.

## 3. Results

### 3.1. Frying Oil Degradation Control Parameters

Previous studies demonstrated that as the frying process advances, oil degrades and an increase in more polar compounds is observed. These compounds act as a surfactant, increasing the contact between the food and the frying oil, favouring oil uptake. Moreover, the increase in oil viscosity due to polymerization reactions during oil degradation implies a higher oil uptake, as large amounts can more easily stick in the crust cavities of the fried food [22,23]. In order to avoid oil uptake interferences due to oil degradation, the polar compounds, iodine value, degradation products such as conjugated di- and trienes, and viscosity of the frying oil were analysed after each experiment, and the oil was discarded when it reached the limits shown in Table 1.

Kim et al. [24] also observed a correlation between the fatty acid composition and the rheological behaviour of an oil. A decrease in the oil viscosity was distinctly observed with an increasing proportion of 18:2 fatty acids and a decreasing proportion of 18:1 fatty acids, which indicated more double bonds in the chain. In the present study, high-oleic sunflower oil was used due to its higher thermal stability than conventional sunflower oil. In Table 2, the fatty acid composition of frying oil and fried mushrooms is given. Monounsaturated fatty acids (MUFA) were the major components in fresh oil. As mushrooms are a low-fat product with only 0.34 g fat/100 g edible portion [25], the fatty acid profile of fried mushrooms was similar to that of frying oil and no differences in fatty acid profile due to the edible coating were detected.

### 3.2. Moisture and Oil Content of Uncoated Samples

Oil uptake in fried products is influenced by different factors such as oil quality, frying temperature, frying time, pre-treatments, and food composition [7,26]. The mechanism of oil uptake involves heat transfer, surface water evaporation, and the movement of oil droplets into the resulting gaps. Thus, when the water losses during frying are reduced, the oil uptake also decreases [7]. It has also been observed that the final oil pickup is not a direct function of porosity but depends, in an intricate manner, on the amount of oil available at the surface, the connectivity of cells, and the size of path sections [27].

Results for the final oil content in fried uncoated mushrooms after different frying conditions are shown in Table 3. They are in accordance with previous studies [7], as a lower final moisture content, due to the dehydration process of the surface, implies a higher oil content. It was also observed that, for the same frying time, the thickness of the product is more influential than the frying oil temperature. Increasing by 30 °C the frying oil temperature, from 150 °C to 180 °C, resulted in an 8.7% higher final oil content, while reducing by 4 mm the sample thickness, from 6 to 2 mm, made the increase 37.6%. This is also related to the greater surface dehydration due to the higher surface/volume ratio the smaller the thickness of the sample.

Furterhmore, the tissue structure size, shape, and initial moisture content of the surface of the food determine the oil uptake during the frying process [2].

Mushrooms are a food product with high moisture (92.45%) and low content of fat, protein, and carbohydrates [25]. Hershko and Nussinovitch (1998) observed that mushrooms have a unique structure in comparison with other fruits and vegetables. Although generally they are seen as a spongy material, the structure and porosity vary if the specimen is observed from the direction of its pileus (cap) or from its velum (veil) and lamellae (gills). From the top, the structure is composed of large holes and is the outcome of branched interconnected hypha filaments of the fungi, which are the major structure of the stem. The hyphal membrane is composed of hemicellulose, cutin, and glycogen. The membrane also contains hydrophobic materials and sterols [28]. 

**Figure 1:**
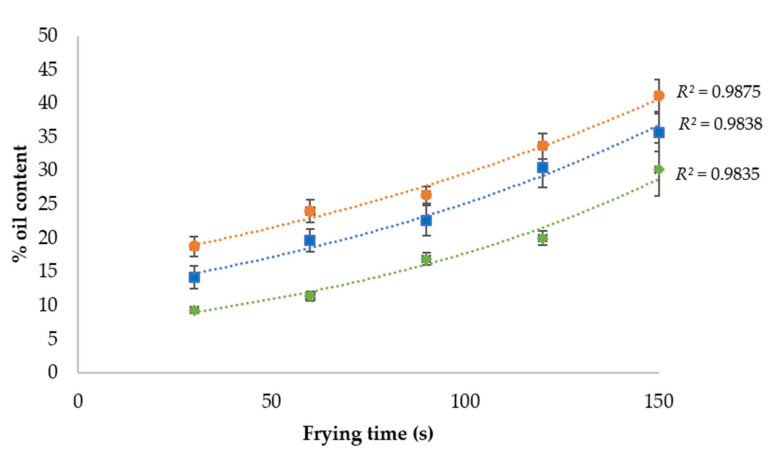
Uncoated mushroom slices’ oil capture kinetics during frying at 170 °C. 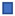 Whole mushroom slice; 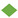 mushroom stem; 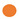 mushroom cap.

Oil capitation kinetics during the frying process in different parts of the mushroom are shown in Figure 1. It could be observed that the oil uptake kinetics fitted (*R*^2^ = 0.9838) to an exponential behaviour in both the whole mushroom and each of its separate parts. As can be observed in Figure 1, the oil content increases the longer the frying time is. Moreover, oil uptake was higher in the cap than in the stem by approximately 12.5%, independently of the moment of the frying process. This could be explained by the higher porosity induced by the holes and their interconnections observed in the cap structure.

### 3.3. Edible Coating Rheology

Two different edible coatings, based on methyl cellulose (MC) and kappa-carrageenan (K-c), were studied. Both were also applied at different temperatures. MC was applied at 15 °C, 30 °C, and 40 °C, while K-c was applied at 30 °C and 40 °C.

Methyl cellulose is a cellulose ether derivative with water solubility at low temperatures and thermoreversible gelation upon heating. Gelation upon heating is due to the self-assembly into fibrils of the MC polymer chains, with a remarkably consistent mean diameter, largely independent of the polymer concentration, molecular weight, and temperature of gelation. This fibrils formation starts over 50 °C [29]. 

Carrageenan is a hydrocolloid polysaccharide that belongs to the family of hydrophilic linear sulphated galactans [30]. Carrageenan, similar to other hydrophilic polymers and polyelectrolytes, has been used as a film-forming solution to control and preserve the texture, flavour, and shelf life of foods. These are employed as gelling agents or thickeners due to their water solubility and ability to increase the viscosity of the continuous (aqueous) phase [31].

**Figure 2:**
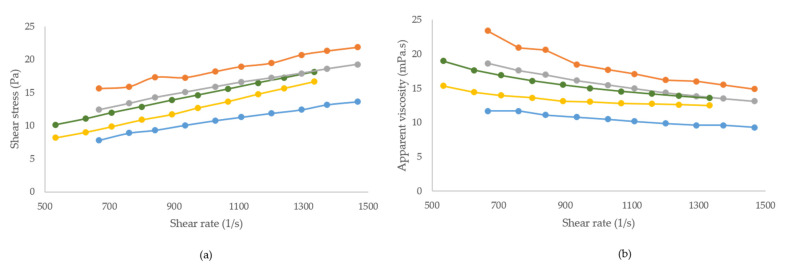
Rheological properties of edible coatings at different temperatures. (**a**) Flow curve; (**b**) apparent viscosity curve. 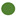 MC 15: methylcellulose at 15 °C; 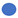 MC 30: methylcellulose at 30 °C; 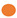 MC 40: methylcellulose at 40 °C; 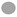 K-c 30: kappa-carrageenan at 30 °C; 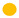
K-c 40: kappa-carrageenan at 40 °C.

In Figure 2, the rheological properties of both edible coatings’ solutions at different temperatures below the gelation point are shown. Viscosity decreased with increasing shear rate, revealing a shear thinning pattern (Figure 2B). This behaviour was previously reported elsewhere for MC and other natural polymers, especially at larger shear rates [32,33]. 

Data from the flow curves were well fitted by the Power Law model (*R*^2^ = 0.9801–0.9993). The consistency index (Keq) and flow index (n) for the different edible coating solutions are given in Table 4. Flow index values (<1) in all cases showed pseudoplastic behaviour. Temperature indicated the apparent viscosity and consistency index. Both parameters decreased as the temperature increased, except for MC-40. Moreira et al. (2017) previously observed similar behaviour in methyl cellulose systems at temperatures under 35 °C [32]. In our results, the apparent viscosity and consistency index of the MC solution were higher at 40 °C, probably because at this temperature molecules interact during the onset of gelation. This gelation behaviour was not observed in kappa-carrageenan solutions, in which aggregation of its double helices increases at lower temperatures and, consequently, its apparent viscosity and consistency index increase [34].

### 3.4. Edible Coating Pick-Up

The measurement of batter pick-up is important as it informs us about the amount of edible coating that remains on the sample. During dip coating of a sample, liquid film thickness is defined by the coating solution properties such as the viscosity and density, and by the process draining time of the coating solution [35]. The analysis of the pick-up of both edible coatings at different temperatures is summarised in Table 5. Pick-up values ranged from approximately 12% to 18%. No significant differences were found in the pick-up of MC-coated samples, independently of the solution’s temperature, while the K-c coating applied at 30 °C showed significant higher values of pick-up than the 40 °C K-c coating.

### 3.5. Moisture and Oil Content of Coated Samples

The moisture and oil content of fried coated samples are shown in Table 5. Both types of edible coating achieved a significant reduction of moisture losses during frying. In the stem, all the edible coating dipping temperatures studied resulted in a similar grade of protection against dehydration, with moisture losses being 12–18% less than for uncoated mushrooms. In the cap, the edible coating led to a 6–14% greater retention of moisture compared to uncoated samples. These results are in accordance with previous studies that observed enhanced moisture retention as a result of a strong interaction due to hydrogen bonding between water molecules when hydrocolloid coatings are applied [36].

Regarding the final oil content, the tendency to absorb significantly more oil in the cap than in the stem remained for all the coated samples, independent of the edible coating and dipping temperature used. Both coating solutions were significantly effective at reducing the oil uptake. Methyl cellulose-coated samples absorbed 10–15% less oil in the stem and 19–22% less in the cap than uncoated mushrooms, with both MC samples dipped at 40 °C showing a lower final oil content. Our results are in accordance with previous studies that indicated that the MC edible coating is one of the most effective hydrocolloids for reducing oil uptake [15,37]. However, in those studies the MC solution also included sorbitol or glycerine as plasticizer. García et al. (2002) also observed similar reductions (−15%) in oil uptake after frying potatoes when MC was applied without a plasticizer [37]. In the case of kappa-carrageenan-coated samples, the reduction in oil uptake was 10–12% in the stem and 10–17% in the cap. With this coating solution, dipping at 30 °C resulted in a higher reduction of dehydration and consequently of oil uptake, especially in the cap. K-c has scarcely been studied as a hydrocolloid for edible coatings to reduce oil uptake. Archana et al. (2016) analysed the effect of carrageenan polysaccharide but in combination with Okra (*Abelmoscus esculentus*) and glycerol as a plasticizer with this aim. They observed higher final moisture and less oil content in coated potato chips in comparison with noncoated samples [38]. 

It should be noted that oil uptake reduction due to edible coatings is slightly higher in the cap than in the stem. This could be caused by the combined effect of the edible coating and the hydrophobin, a hydrophobic protein, which forms hydrophobic rodlet layers on the outer surface of the mushroom. Most of this protein is found within the cap’s outer tissue and none in the gills [39]. Moreover, as previously reported by other authors, the mushroom cap is covered with a monolayer of water molecules that encourages the penetration of the hydrophilic edible coating solution with low viscosity entering the holes [28]. In our study, the dipping process was done immediately after the mushroom was sliced. This fact favours better adhesion and coverage throughout the food by reducing the amount of velum, which is the unique structure that prevents the penetration of liquids in contact with the edible coating.

**Figure 3:**
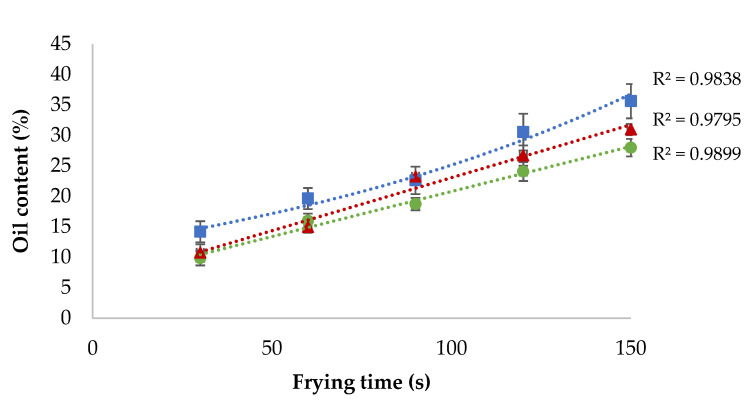
Mushroom slices oil capture kinetics during frying at 170 °C. 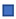 Mushroom, noncoated; 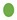 mushroom with methyl cellulose coating; 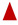 mushroom with kappa-carrageenan coating.

Oil capture kinetics in fried mushroom slices also showed differences if they were coated (Figure 3). As previously shown in uncoated samples (Figure 1), it could be observed that the oil content in coated samples is higher in longer frying times. Oil uptake kinetics fitted (*R*^2^ = 0.9838) to an exponential behaviour when the sample was uncoated. However, the oil uptake kinetics fitted better to a linear behaviour (*R*^2^ = 0.9795 and *R*^2^ = 0.9899, respectively) if the mushrooms were coated with a K-c or MC solution. This could be explained by the low rate of dehydration caused by the hydrocolloid coating. As the frying time advances, water evaporation increases, resulting in more gaps that will be refilled with oil droplets. However, as both hydrocolloid edible coatings, MC and K-c act as moisture retention barriers, the decreasing water evaporation rates and a small number of empty gaps for oil alter the oil uptake kinetics.

## 4. Conclusions

Parameters such as frying conditions, slice thickness, and food structure are determinants of oil uptake during the frying process. Higher temperature, thinner slices, and a more porous structure, such as in a mushroom’s cap, resulted in lower final moisture and higher final oil content. Oil uptake kinetics during frying fitted to an exponential behaviour, both in the whole mushroom and in each of its component parts. 

Both hydrocolloid suspensions, methyl cellulose and kappa-carrageenan, used as edible coatings were effective at reducing the moisture evaporation and, consequently, the oil uptake independently of the hydrocolloid temperature. A reduction of 10–22% in the final oil content was reached. Oil uptake reduction was slightly higher in the cap than in the stem due to its more porous structure. 

Oil uptake kinetics were modulated by the hydrocolloid edible coating, fitting better to a linear behaviour when samples were coated.

Adjusting the oil frying temperature and food parameters such as slice thickness are easy strategies to reduce the oil uptake in fried products. Moreover, the inclusion of methyl cellulose or kappa-carrageenan as an edible coating could be of interest to industrial manufacturers and restaurants hoping to achieve this objective. Therefore, the wider application conditions of hydrocolloids, such as concentration, temperature, dwell time before application, etc., may be the subjects of future studies.

## Figures and Tables

**Table 1:** Oil frying parameters and limits to change.

Polar compounds (%)	15%
Iodine value	87
Conjugated dienes (abs)	<0.800
Conjugated trienes (abs)	<0.600
Viscosity (mPas)	67–70

**Table 2:** Content of the main fatty acids (g/100 g FA) in frying oil and fried mushrooms.

	Raw Mushrooms (USDA) [25]	Frying Oil	Fried Mushrooms
C14:0	0.00	0.05 ± 0.01	0.05 ± 0.00
C16:0	0.04	4.57 ± 0.02	4.58 ± 0.01
C16:1	0.00	0.14 ± 0.04	0.13 ± 0.00
Heptadecanoate	-	0.03 ± 0.01	0.02 ± 0.00
Heptadecenoic	-	0.04 ± 0.01	0.04 ± 0.00
C18:0	0.01	3.84 ± 0.02	3.84 ± 0.01
C18:1 trans	0.16	0.06 ± 0.02	0.06 ± 0.01
C18:1 cis	-	71.44 ± 0.15	71.50 ± 0.15
C18:2 cis-trans	-	0.08 ± 0.00	0.08 ± 0.00
C18:2 trans-cis	-	0.06 ± 0.00	0.06 ± 0.00
C18:2	0.00	17.79 ± 0.19	17.76 ± 0.00
C20:4	-	0.32 ± 0.00	0.32 ± 0.00
C18:3 + C20:1	-	0.27 ± 0.01	0.27 ± 0.01
C22:0	-	0.97 ± 0.00	0.97 ± 0.00
C24:1	-	0.32 ± 0.01	0.31 ± 0.00
Ʃ SFA		9.76 ± 0.06	9.76 ± 0.04
Ʃ MUFA		71.65 ± 0.20	71.69 ± 0.16
Ʃ PUFA		18.52 ± 0.20	18.48 ± 0.17
Ʃ Trans		0.20 ± 0.02	0.19 ± 0.01

**Table 3:** Effect of frying temperature and slice thickness in final oil and moisture content of whole uncoated mushroom slices.

Frying Time (s)	Frying Temperature (°C)	Thickness (mm)	Oil (%)	Moisture (%)
150	150	4.0 ± 0.2	31.7 ± 2.2 a	46.4 ± 1.0 a
150	170	4.0 ± 0.2	35.6 ± 2.8 b	33.8 ± 3.9 b
150	180	4.0 ± 0.2	40.4 ± 2.8 c	27.7 ± 4.9 c
150	170	2.0 ± 0.2	63.0 ± 3.7 d	3.3 ± 1.2 d
150	170	4.0 ± 0.2	35.6 ± 2.8 b	33.8 ± 3.9 b
150	170	6.0 ± 0.2	25.4 ± 3.3 e	52.4 ± 1.9 e

Results expressed as mean values ± standard deviation. Different letters in a column indicate significant differences (*p* ≤ 0.05).

**Table 4:** Rheological characteristics of the different edible coating solutions fitted to the Power Law model.

	Consistency Index (Keq)	Flow Index (n)	R2
MC 15	189.60 ± 84.75 a	0.64 ± 0.05 a	0.9988
MC 30	90.93 ± 16.19 b	0.69 ± 0.06 a	0.9963
MC 40	856.84 ± 137.09 c	0.45 ± 0.10 b	0.9801
K-c 30	346.50 ± 72.00 d	0.55 ± 0.06 b	0.9993
K-c 40	48.39 ± 7.58 e	0.81 ± 0.05 c	0.9842

MC 15: methylcellulose at 15 °C; MC 30: methylcellulose at 30 °C; MC 40: methylcellulose at 40 °C; Kappa 30: kappa-carrageenan at 30 °C; Kappa 40: kappa-carrageenan at 40 °C. Mean values ± standard deviations. Different letters in each column indicate significant differences (*p* < 0.05).

**Table 5:** Differences in the pick-up, moisture, and final oil content of samples prepared with different edible coatings, after frying at 170 °C for 150 s.

	Pick-Up (%)	Moisture (%)			Oil (%)		
		Stem	Cap	*p*	Stem	Cap	*p*
WH	-	36.73 ± 6.13 a	30.44 ± 1.85 a	0.0564	30.13 ± 3.91 a	41.61 ± 2.21 a	0.0003
MC 15	15.07 ± 1.90 a	48.35 ± 0.73 b	44.50 ± 2.74 b,c	0.0443	20.00 ± 2.52 b	22.57 ± 1.74 b,e	0.0438
MC 30	15.13 ± 1.90 a	50.82 ± 1.88 b	42.79 ± 3.74 b	0.0154	19.24 ± 1.63 b	21.07 ± 0.77 b	0.0195
MC 40	15.18 ± 1.71 a	54.50 ± 0.61 b	44.75 ± 3.32 b,c	0.0086	15.09 ± 1.86 c	19.90 ± 0.76 c	0.0065
K-c 30	17.68 ± 2.65 b	50.60 ± 5.94 b	41.66 ± 5.03 b,c	0.0626	17.96 ± 0.61 b,c	24.57 ± 1.60 e	<0.0001
K-c 40	12.23 ± 1.82 c	53.04 ± 3.89 b	36.24 ± 2.71 c	0.0006	20.53 ± 2.13 b	31.19 ± 1.32 d	<0.0001

WH: without coating; MC 15: methyl cellulose at 15 °C; MC 30: methyl cellulose at 30 °C; MC 40: methyl cellulose at 40 °C; K-c 30: kappa-carrageenan at 30 °C; K-c 40: kappa-carrageenan at 40 °C. Mean values ± standard deviations. Different letters in each column indicate significant differences (*p* ≤ 0.05). The *p*-value indicates a statistical level of significance between stem and cap final moisture and oil content differences.
